# Identification and Characterization of the *DMRT11E* Gene in the Oriental River Prawn *Macrobrachium nipponense*

**DOI:** 10.3390/ijms20071734

**Published:** 2019-04-08

**Authors:** Yabing Wang, Shubo Jin, Hongtuo Fu, Hui Qiao, Shengming Sun, Wenyi Zhang, Sufei Jiang, Yongsheng Gong, Yiwei Xiong, Yan Wu

**Affiliations:** 1 Wuxi Fisheries College, Nanjing Agricultural University, Wuxi 214081, China; wang113013@126.com; 2 Key Laboratory of Freshwater Fisheries and Germplasm Resources Utilization, Ministry of Agriculture, Freshwater Fisheries Research Center, Chinese Academy of Fishery Sciences, Wuxi 214081, China; jinsb@ffrc.cn (S.J.); qiaoh@ffrc.cn (H.Q.); sunsm@ffrc.cn (S.S.); zhangwy@ffrc.cn (W.Z.); jiangsf@ffrc.cn (S.J.); gongys@ffrc.cn (Y.G.); xiongyw@ffrc.cn (Y.X.); wuy@ffrc.cn (Y.W.)

**Keywords:** *Macrobrachium nipponense*, *DMRT11E*, temporal and spatial expression, in situ hybridization, RNA interference

## Abstract

The doublesex and mab-3 related transcription factor (*DMRT*) gene family involvement in sex development is widely conserved from invertebrates to humans. In this study, we identified a DM (*Doublesex/Mab-3*)-domain gene in *Macrobrachium nipponense*, which we named *MniDMRT11E* because it has many similarities to and phylogenetically close relationships with the arthropod *DMRT11E*. Amino acid alignments and structural prediction uncovered conservation and putative active sites of the DM domain. Real-time PCR analysis showed that the *MniDMRT11E* was highly expressed in the ovary and testis in both males and females. Cellular localization analysis showed that *DMRT11E* was mainly located in the oocytes of the ovary and the spermatocyte of the testis. During embryogenesis, the expression level of *MniDMRT11E* was higher at the cleavage stage than at other stages. During the different stages of ovarian development, *MniDMRT11E* expression gradually increased from OI to OIII and decreased to the lowest level at the end of OIV. The results indicated that *MniDMRT11E* probably played important roles in embryonic development and sex maturity in *M. nipponense*. *MniDMRT11E* dsRNA injection also significantly reduced vitellogenin (*VG*) expression and significantly increased insulin-like androgenic gland factor (*IAG)* expression, indicating a close relationship in gonad development.

## 1. Introduction

The oriental river prawn, *Macrobrachium nipponense*, is an economically important freshwater prawn and is widely farmed in China, with an annual production of almost 240,739 tons in 2017 [1]. Male oriental river prawns grow faster than females. The average size of the male commodity is 2-2.5 times that of females. All male cultures will help to increase yields and economic value. The development of an all-male culture in *M. nipponense* is based on sexual control technology. Therefore, it is important to identity sex-determining genes and their regulatory mechanism.

Various sex determination methods in animals are the focus of current research. Sex-determining methods in insects that are close to crustaceans have recently been studied, especially in model species such as *Drosophila* [2,3]. The sex determination mechanism of crustaceans is relatively complex and impacted by environmental factors. At present, there are limited studies, and most species-related studies are still lacking. Insulin-like androgenic gland factor (*IAG*) is a unique gene in crustaceans that is specifically expressed in the androgenic gland (AG), and also plays an important role in sex differentiation, but the specific molecular mechanism is not yet clear [4,5,6,7,8].

The molecular pathways of sex determination and differentiation are highly diverse in the sexual reproduction of animals. This has been interpreted as a result of the rapid evolution of the genes involved in the process [9]. However, the mechanism of sex determination is different in different animal lineages [10]. In insects, cell-based autonomic and splicing-based mechanisms have been shown to be responsible for sex dimorphism; while in mammals, gonadal-dependent endocrine control features can differentially determine and maintain male and female phenotypes [11]. Although different upstream mechanisms are involved in sex determination, downstream-related genes appear to be more conservative [12]. Among the most widely studied genes are the doublesex and mab-3 related transcription factor (*DMRT*) gene families [13]. *DMRT* proteins share a distinctive zinc-finger DNA binding motif termed the DM domain [14,15,16]. *Doublesex* (*dsx*), the first gene, was originally discovered at the bottom of the *Drosophila melanogaster* sex determination cascade [17]. Another DM domain gene, mab-3, is required for the synthesis of vitellin and the male-specific lineage in *Caenorhabditis elegans* [18]. Members of the DMRT gene family are widely represented in the animal kingdom. For example, *DSX* is only found in arthropods, *DMRT1* is only found in vertebrates, and *DMRT6-9* are only found in mammals [19].

The *DMRT* gene has been studied for decades in vertebrates, nematodes, and insects; however, interest in crustaceans is comparatively recent [10]. The first crustacean DM-domain gene from *Eriocheir sinensis* represents a testis-specific expression pattern [9]. In addition, the *Daphnia magna*, *DmaDsx1*, exhibits a pattern of sexual dimorphism and is responsible for male-specific development [20]. Two *DMRT* genes were also found in *Macrobrachium rosenbergii*, which is closely related to *M. nipponense*, and one of these was found to be related to the expression of *IAG* [10]. In crustaceans, genes encoding the DM-domain (DM-domain genes) may play a related role in sex determination [9,10,20].

In decapod crustaceans, as in vertebrates, the vitellogenesis oocyte is a huge growing oocyte with a cytoplasm filled with eosinophilic cells. Early vitellogenesis oocytes contain yolk granules and blocks deep into the yolk, and the nucleus is located in the center of the oocytes. The mature period of the oocytes, with the greatest volume, is in the late vitellogenesis stage. The nucleus surrounds the oocyte and, eventually, the germinal vesicle breaks down and the nuclear membrane disappears. The egg around the follicular cell layer then becomes extremely thin [21]. The testes contain many seminiferous tubules. Each small tubule is made up of germinative epithelium and a tubule liner. The germinative epithelium produces a variety of spermatogeni cell areas. The vas deferens can be morphologically divided into three regions: the anterior; the median, which is highly convoluted; and the posterior. The vas deferens is composed of columnar epithelium surrounded by striated muscle. Two types of cells, high columnar cells and low columnar cells, which can secrete spermatophore matrixes, can be distinguished in the epithelium of the vas deferens. The terminal ampulla is highly muscularized [9,22,23]. The AG is located at the end of the vas deferens and the ejaculatory bulb [24]. The AG is a small lobular endocrine organ [25]. The AG of *Macrobrachium* possesses three cell types: Type I cells are small polygonal shaped-cells, and stain strongly with hematoxylin–eosin (H&E); type II cells are slightly larger, and stain lightly with H&E; and type III cells are similar in size and shape to type I cells, but the cytoplasm is unstained [25].

In this study, we isolated and characterized a gene with a DM domain in *M. nipponense*. Homology comparison and phylogenetic analysis of putative proteins indicate that it is a member of the *MniDMRT11E* subfamily. Tissue distribution analysis of *DMRT11E* transcription levels in the gonads was significantly higher than in other tissues. In addition, the knockdown of *MniDMRT11E* had a significant impact on the transcriptional expression level of *MnIAG* and *MnVG*.

## 2. Results

### 2.1. Molecular Cloning and Structural Analysis of DMRT11E Gene

**Figure 1:**
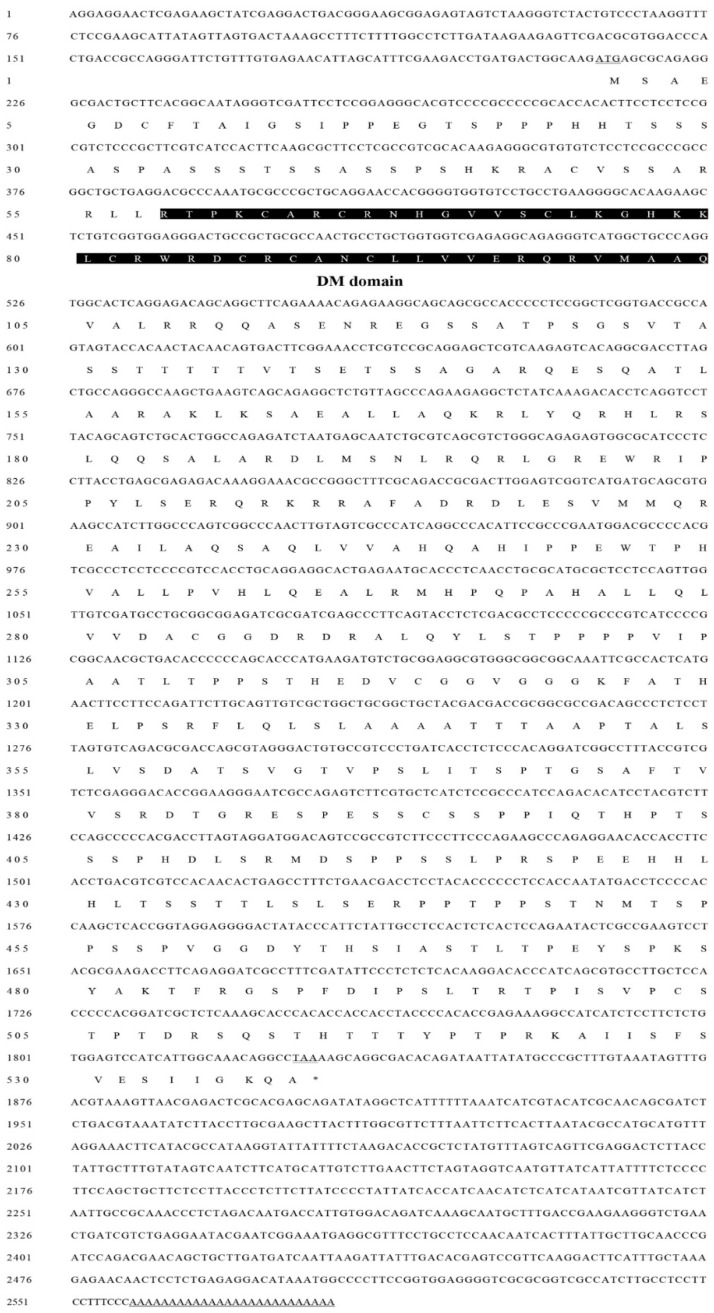
Nucleotide and deduced amino acid sequence of *Macrobrachium nipponense DMRT11E* gene (*MniDMRT11E*) cDNA. 5′ UTR and 3′ UTR are listed with lowercase letters. ORF are shown by capital letters. The translation start codon (ATG) and termination codon (TAA) are underlined in the figure. The DM domain is marked with shadow.

**Figure 2:**
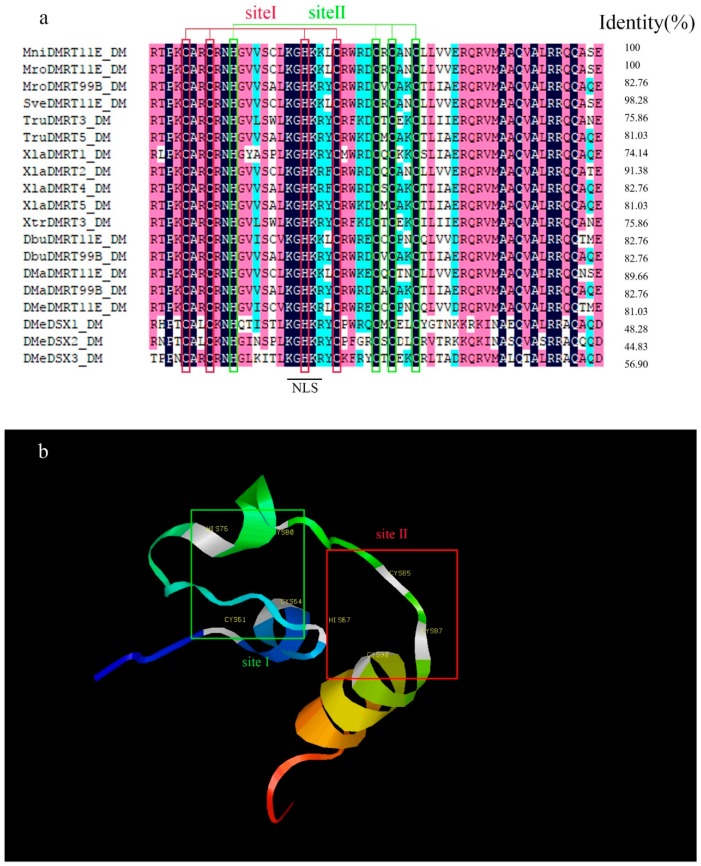
Amino acid alignment and structure prediction of DM domains of *MniDMRT11E*. (**a**) Homologous comparison of amino acid sequences of DM domains from various *DMRT* proteins. Consensus amino acids are highlighted. The right columns show the identities of MniDMRT11E_DM with other *DMRT* proteins (*MniDMRT11E*_DM (In this study), *MroDMRT11E*_DM (*Macrobrachium rosenbergii* AHI47024.1), *MroDMRT99B*_DM (*Macrobrachium rosenbergii* AHI47025.1), *SveDMRT11E*_DM (*Sagmariasus verreauxi* ARK36622.1), *TruDMRT3*_DM (*Takifugu rubripes* NP_001033034.1), *TruDMRT5*_DM (*Takifugu rubripes* NP_001033039.1), *XlaDMRT1*_DM (*Xenopus laevis* NP_001089969.1), *XlaDMRT2*_DM (*Xenopus laevis* NP_001089725.1), *XlaDMRT4*_DM (*Xenopus laevis* AAV66322.1), *XlaDMRT5*_DM (*Xenopus laevis* NP_001089148.1), *XtrDMRT3*_DM (*Xenopus tropicalis* NP_001243149.1), *DbuDMRT11E*_DM (*Drosophila busckii* ALC49006.1), *DbuDMRT99B*_DM (*Drosophila busckii* ALC47170.1), *DMaDMRT11E*_DM (*Daphnia magna* BAG12871.1), *DMaDMRT99B*_DM (*Daphnia magna* BAG12873.1), *DMeDMRT11E*_DM (*Drosophila melanogaster* NP_511146.2), *DMeDSX1*_DM (*Drosophila melanogaster* NP_731197.1), *DMeDSX2*_DM (*Drosophila melanogaster* NP_731198.1), *DMeDSX3*_DM (*Drosophila melanogaster* NP_524272.4)). Putative nuclear localization signals (NLSs) are underlined. (**b**) 3D-structures of *MniDMRT11E* predicted by I-TASSER. Green rectangle (site I) and red rectangle (site II) represent the residues of two intertwined Zn^2+^-binding sites.

**Figure 3:**
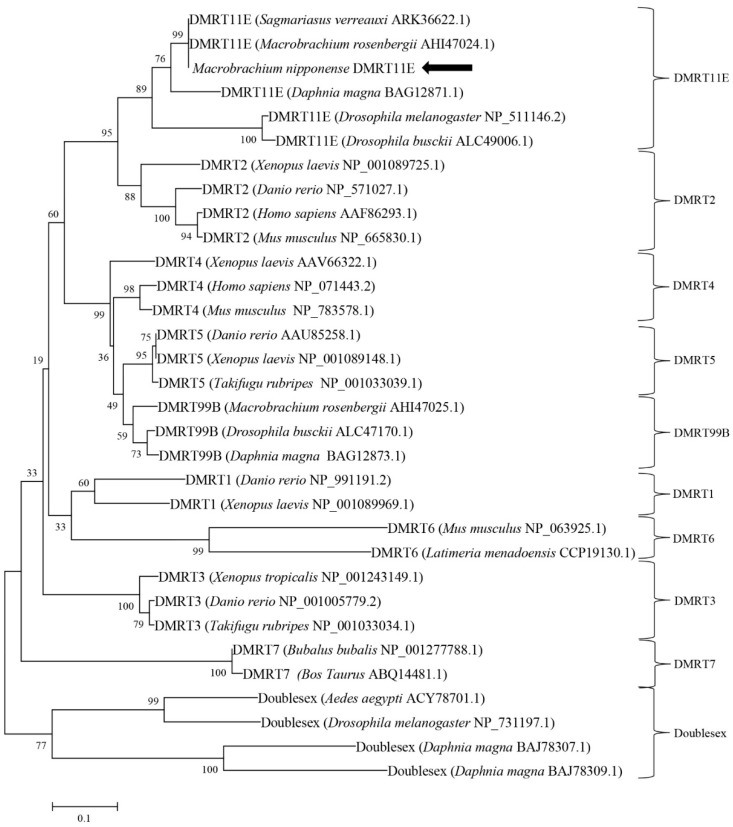
Phylogenetic analysis of DM family members. The diagram was generated by the neighbor-joining method using the MEGA 5.1 program. Bootstrapping replications were 1000. GenBank accession numbers are in brackets.

The full length of the cloned single *DMRT* cDNA is 2585 bp (GenBank accession number, MH636338) and encodes 542 amino acids. The 5′-untranslated region (UTR) is 200 bp long, and the 3′-UTR is 756 bp long. The termination codon and polyadenylation signal located on the 3′-RACE PCR product revealed the integrity of the sequences we cloned (Figure 1). The sequence obtained from the 3’-RACE PCR product contained a stop codon. The homologous comparison (Figure 2a) and phylogenetic analysis (Figure 3) revealed it was most closely related to the *DMRT11E* subfamily of the *DMRT* protein family; therefore, we refer to the gene as *MniDMRT11E*. Blast searches showed that the protein structure of *MniDMRT11E* was similar to that of the following species: *Macrobrachium rosenbergii* (*DMRT11E*, 93%), *Sagmariasus verreauxi* (*DMRT11E*, 76%), *Daphnia magna* (DMRT11E, 53%), *Amazona aestiva* (*DMRT2* 48%), and *Fundulus heteroclitus* (*DMRT2*, 47%).

In MniDMRT11E, the two intertwined Zn^2+^ -binding sites (sites I C61/C64/H76/C80 and sites II H67/C85/C87/C90) are necessary for DNA binding (Figure 2). The DM domain also contains a conserved nuclear localization signal (NLS) (Figure 2a). DM domains are more conserved in *DMRT11Es* (81.03–100%) and are similar to vertebrate *DMRT2s* (91.4%).

### 2.2. Tissue-Specific Expression Patterns of MniDMRT11E

**Figure 4:**
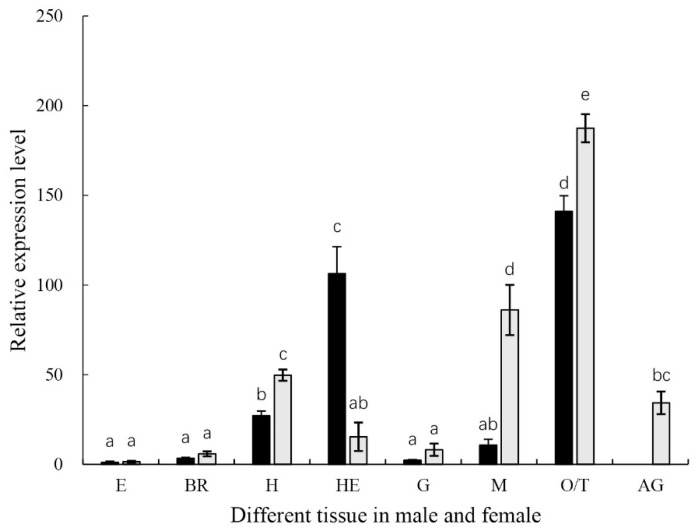
The expressions of *MniDMRT11E* revealed by qPCR in different tissues. The amount of two genes’ mRNA was normalized to the β-actin transcript level. Data are shown as means ± SD (*n* = 4 prawns). E: eyestalk; BR: brain; H: heart; HE: hepatopancreas; G: gill; M: muscle; O: ovary; T: testis; AG: abdominal ganglia. Statistical analyses were performed with one-way ANOVA analysis. The post-hoc test following ANOVA analysis used LSD. Bars with different letters were considered significant at *p* < 0.05. Values are means ± standard error of the mean (SE) for quadruplicate samples.

The expression levels were analyzed in adult prawn tissues by qPCR, and the results showed that *MniDMRT11E* mRNA was distributed in all tissues (Figure 4). *MniDMRT11E* was highest in the testis (*p* < 0.05), followed by the ovary (*p* < 0.05). *MniDMRT11E* was expressed more highly in the male hepatopancreas than in the female. However, the level of expression in the muscles was the opposite.

### 2.3. Expression of the MniDMRT11E Gene During Embryo Stages

**Figure 5:**
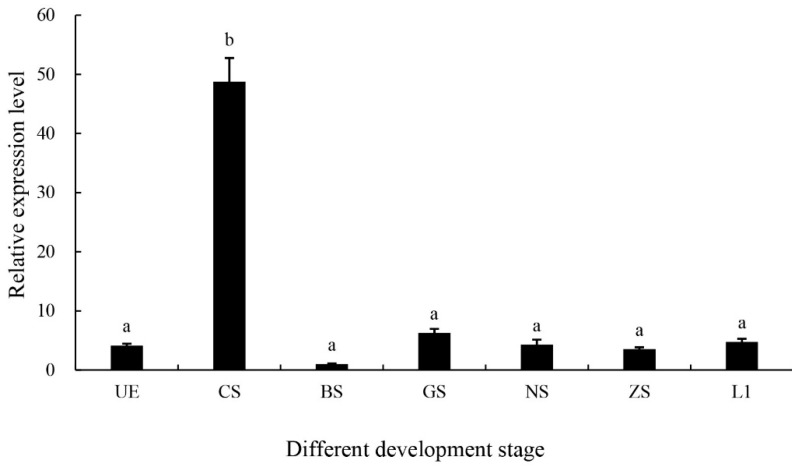
The expressions of *MniDMRT11E* revealed by qPCR in different embryo stages. The amount of *MniDMRT11E* mRNA was normalized to the β-actin transcript level. UE: unfertilized egg; CS: cleavage stage; BS: blastula stage; GS: gastrula stage; NS: nauplius stage; ZS: zoea stage; L1: the first day larvae after hatching. Data are shown as means ± SD (*n* = 4 prawns). Statistical analyses were performed with one-way ANOVA analysis. The post-hoc test following ANOVA analysis used LSD. Bars with different letters were considered significant at *p* < 0.05. Values are means ± standard error of the mean (SE) for quadruplicate samples.

The *MniDMRT11E* expression pattern was analyzed by qPCR in different developmental stages (from the cleavage stage to the first-day larvae after hatching) (Figure 5). A relatively high level of *MniDMRT11E* expression was observed in the cleavage stage of embryos (*p* < 0.05). The expression at other developmental times was not significant (*p* > 0.05).

### 2.4. Expression of the MniDMRT11E Gene in Different Developmental Stages of the Ovaries

**Figure 6:**
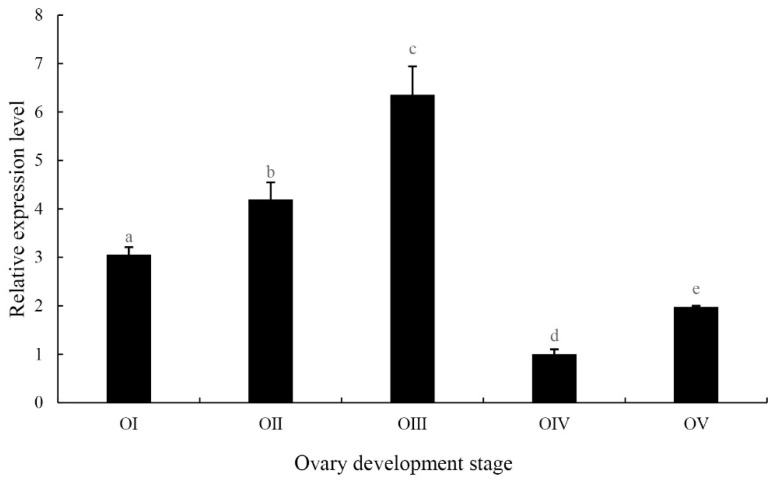
Quantitative analysis of *MniDMRT11E* transcripts using real-time PCR in different development stages of ovaries. O I: undeveloped stage; O II: developing stage; O III nearly-ripe stage; O IV: ripe stage; O V: spent stage. Data are shown as means ± SD (*n* = 4 prawns). Bars with different letters were considered significant at *p* < 0.05. Statistical analyses were performed with one-way ANOVA analysis. The post-hoc test following ANOVA analysis used LSD. Values are means ± standard error of the mean (SE) for quadruplicate samples.

Figure 6 shows the expression patterns of *MniDMRT11E* throughout the reproductive cycle, assessed by qPCR. The results confirmed a regular expression throughout the maturation of the ovary. The *MniDMRT11E* transcript level was low at the beginning of the reproductive cycle (stage I, undeveloped stage) and increased to a maximum at the nearly-ripe stage (stage III). Beyond this stage, the level fell to the lowest level at the ripe stage (stage IV). This implies that *MniDMRT11E* may play a role in reproductive development.

### 2.5. Localization of the MniDMRT11E Gene in the Gonad

**Figure 7:**
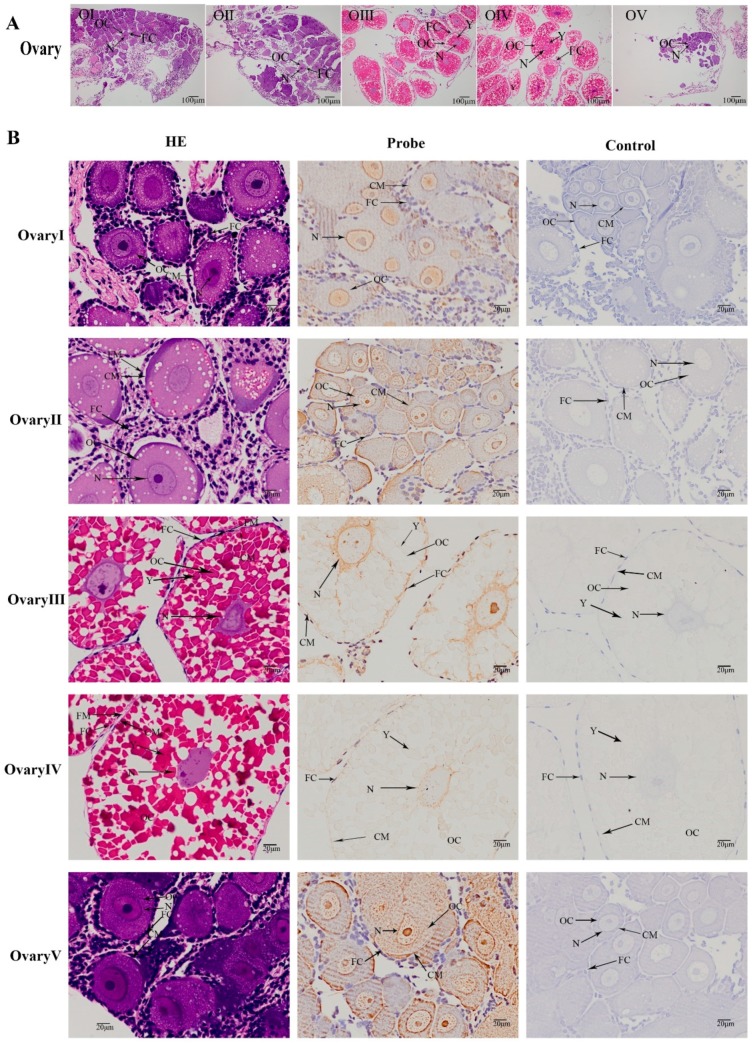
(**A**) Photograph of *M. nipponense* ovary in ovarian cycle. OG: oogonium; OC: oocyte; N: nucleus; CM: cytoplasmic membrane; Y: yolk granule; FC: follicle cell. (**B**) Histological section of ovary at different ovary stages of *M. nipponense*. OG: oogonium; OC: oocyte; N: nucleus; CM: cytoplasmic membrane; Y: yolk granule; FC: follicle cell; FM: follicle membrane. (**A**) scale bars: 100×. (**B**) scale bars: 400×.

According to previous results [26] and ovary color observation, the ovarian cycle of prawns was divided into five stages: transparent (undeveloped, oogonium proliferation, Stage I), yellow (developing, primary vitellogenesis, Stage II), light green (nearly-ripe, secondary vitellogenesis, Stage III), dark green (ripe, vitellogenesis termination, Stage IV), and gray (spent, Stage V) (Figure 7A). The cellular localization of *MniDMRT11E* was examined in different development stages of ovaries by in situ hybridization. ISH revealed a *MniDMRT11E* signal in the same locations in all the oocytes types (Figure 7B). The signal was visualized in all of the oocyte types, including yolk granules, the nucleus, and the cytoplasmic membrane (Figure 7B). The signal appearance in the cytoplasm was closed to the nucleus.

**Figure 8:**
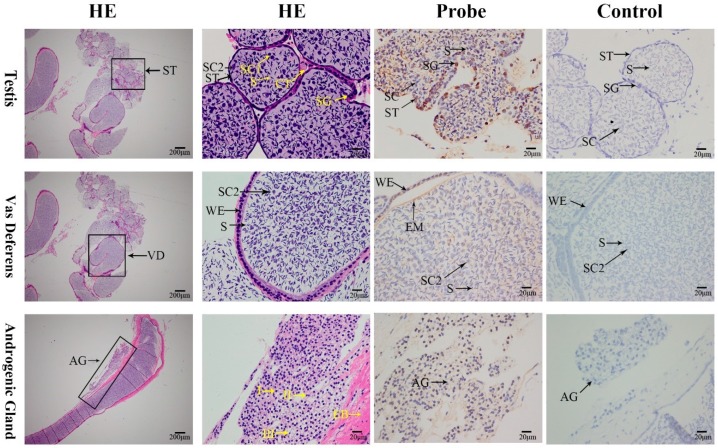
Histological section of testis of *M. nipponense*. CT: collecting tissue; SG: spermatogonium; SC: spermatocyte; SC1: primary spermatocyte; SC2: second spermatocyte; ST: spermatid; S: sperm. WE: wall epithelium; EM: eosinophilic matrix; VD: vas deferens; EB: the ejaculatory bulb; AG: androgenic gland. Within each lobule, there are three types of cells (I, II, and III). The first column: scale bars: 40×, others: scale bars: 400×.

The male reproductive system consists of paired testes, vas deferens, terminal ampulla, and male gonopore. In mature testes, the *MniDMRT11E* mRNA was visualized in spermatogonia during spermatogenesis (Figure 8). In vas deferens, the *MniDMRT11E* mRNA was visualized in the eosinophilic matrix (Figure 8), and in the androgenic gland, the *MniDMRT11E* mRNA was visualized in the nucleus and cell membrane of three glandular cells (Figure 8).

### 2.6. Effects of MniDMRT11E Knockdown on Gonad by RNAi

**Figure 9:**
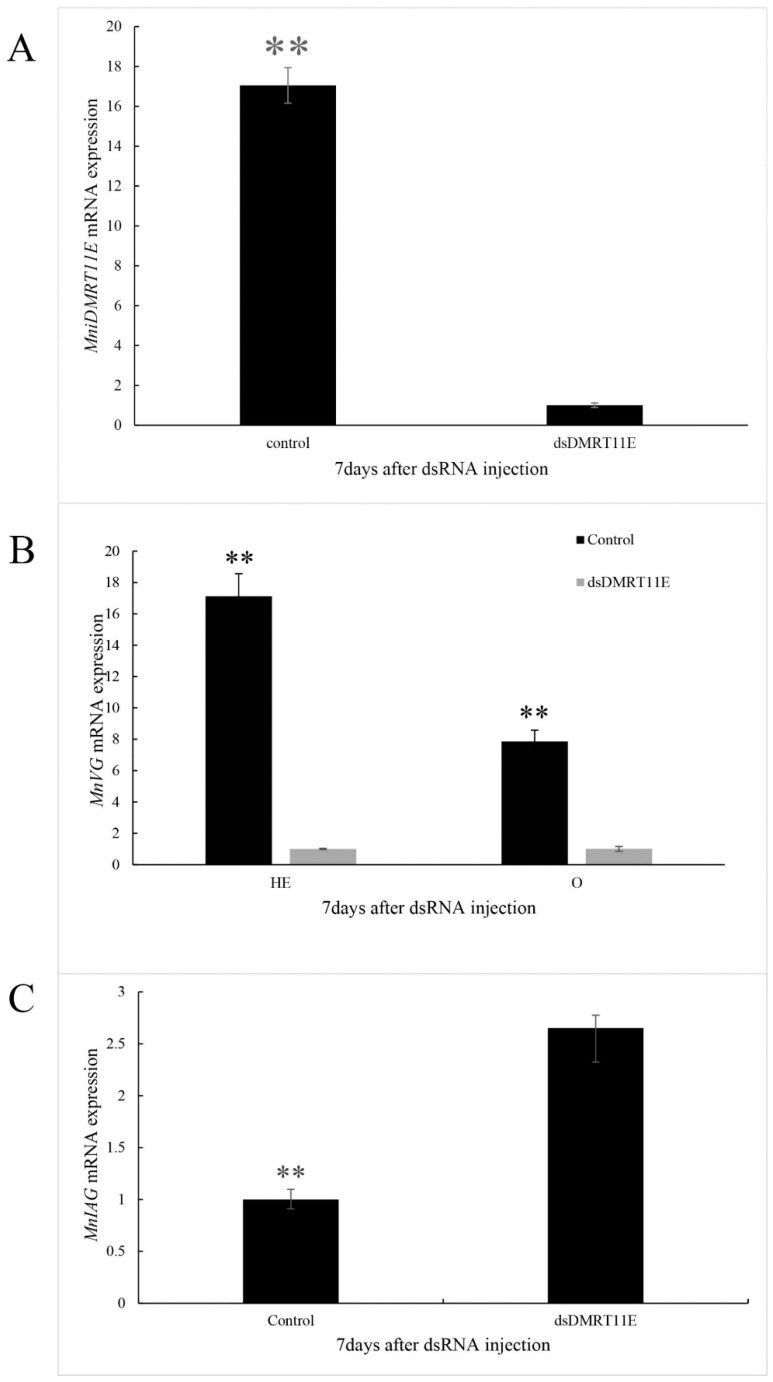
The *MniDMRT11E* expression level (**A**) after RNA interference. Vitellogenin (*VG*) (GenBank: KJ768657) expression level in the ovary and hepatopancreas and (**B**) after RNA interference in female specimens. Insulin-like androgenic gland factor (*IAG*) (GenBank: KF811212) expression level (**C**) in the abdominal ganglia after RNA interference in male specimens. HE: hepatopancreas; O: ovary. Results are expressed as mean ± SEM and significance of comparison is defined as *p* < 0.01 (**) by Student’s *t* tests.

Considering that *MniDMRT11E* exhibits a dimorphic expression pattern, we used RNAi to investigate the role of *MniDMRT11E* function in male/female phenotypic development/maintenance of *M. nipponense*. Intravenous injection of RNAi-mediated knockout with dsRNA was apparently successful, and on the seventh day after injection, *MniDMRT11E* expression was down-regulated by 86% (*p* < 0.01, Figure 9A) compared with control levels. After *MniDMRT11E* RNAi, we observed that the *VG* (female) transcript decreased significantly by 60 % in the ovary and 94% in the hepatopancreas (*p* < 0.01, Figure 9B). However, we observed a nearly two-fold increase in the *IAG* transcript (*p* < 0.01, Figure 9C). These results indicated that *MniDMRT11E* RNAi reduced *VG* accumulation and increased *IAG* accumulation.

## 3. Discussion

*DMRT* or dmrt-like genes have been cloned in several invertebrates, but due to a lack of genomic information, it is difficult to annotate and identify *DMRT* genes in different invertebrate groups [27,28]. In this study, we identified a *DMRT* gene corresponding to *MniDMRT11E* in *M. nipponense* and performed a series of in silico analyses, such as amino acid translation, domain/motif identification and comparison, and phylogenetic analysis, to characterize the *MniDMRT11E* gene [22]. This is the first gene with a DM-domain to be identified in *M. nipponense* and it was shown that the DM domain was well-conserved, as in other species. All *DMRT* amino-acid sequences contain a single conserved DNA-binding motif known as the DM domain. The DM motif was a cysteine-rich DNA-binding domain comprising interwoven CCHC and HCCC Zn^2+^ binding sites and a putative nNLS consisting of KGHKK/R (Figure 2) [29]. Phylogenetic analysis indicated that the *MniDMRT11E* protein clustered with the *DMRT11E* sequences of other species.

The *DMRT11E* gene is usually expressed in a sex-specific manner in many species. In *Drosophila melanogaster*, three DM genes were identified: *DMRT11E*, *DMRT93B*, and *DMRT99B*. Quantitative gene expression analysis in gonads showed that *DMRT11E* is expressed higher in the ovary than in the testis [29]. In *Daphnia magna*, the *DMRT11E* was also highly expressed in the ovary [29]. However, in *Macrobrachium rosenbergii*, *DMRT11E* transcription was prominent in the testis, while much lower in the ovary [10]. In our study, *DMRT11E* transcription was prominent in both the testis and ovary; moderate in hepatopancreas (female) and muscle (male); and much lower in the eyestalk, brain, and gill (Figure 4) (*p*<0.05). However, the levels were much higher in the testis than in the ovary. The expression profile of the *MniDMRT11E* is quite similar to that of *Macrobrachium rosenbergii MroDMRT11E* in the testis [10]. The cellular localization of the *MniDMRT11E* transcripts has also been examined in spermatogonia in the testis and oocyte in the ovary. Together, these findings suggest that *MniDMRT11E* is an important gene in gonad maturity. 

During embryo stages, we founded that the abundance of *MniDMRT11E* mRNA in the cleavage stage is much higher than in other stages (Figure 5). Due to the elaborations in arthropods, the clues to the exact role of *DMRT11E* can be indicated by the function of their vertebrate homologs (*DMRT2s*; Figure 5). In zebrafish, two genes, *DMRT2a* and *DMRT2b*, are present. In adult tissues, the zebrafish *DMRT2a* and *DMRT2b* mRNA is expressed highly in muscle tissues. In different stages of embryos, *DMRT2b* transcripts appear in all stages of embryos, and the level of *DMRT2b* transcripts increases during late development. *DMRT2a* transcripts appeared at the blastula stage and reached a peak at the bud stage immediately before segmentation. *DMRT2a* is necessary for symmetric somite formation, while *DMRT2b* regulates somite differentiation impacting on slow muscle development [30]. In mice, *DMRT2* is expressed in the dermomyotome of developing vertebrate somites [31]. This could explain why *MniDMRT11E* is highly expressed in muscle (male) (Figure 4).

Ovogenesis refers to the process in which primordial oogonium cells develop into oocytes cells and develop into mature eggs. Oogonium cells first mitotically propagate in the ovary, and then enter meiosis to become oocytes. In the current study, different expression patterns were also detected in the ovary. The expression level of *MniDMRT11E* was lower at the beginning of the reproductive cycle (stage I), and then increased and peaked at the oil globules stage (stage III), after which a decrease in levels was observed. In addition, the cellular localization of *MniDMRT11E* s mRNA was visualized in all the oocyte, including yolk granules, the nucleus, and the cytoplasmic membrane (Figure 5). This means it is important for oocyte development.

Among the known invertebrate model species, Caenorhabditis elegans and *Drosophila* have sex-determining genes called mab-3 and doublesex, respectively, which have one common DNA-binding motif in the gate (DM) domain. The DM domain has regulatory relationships with certain genes, such as *VG* and *IAG* [10,29,32,33,34,35,36]. Although the foregoing results suggest a relationship between *MniDMRT11E* and gonad maturity, the precise function of *MniDMRT11E* during gonad maturity remains unclear. RNAi has been helpful in studying more and more crustaceans and revealing the function of novel crustacean genes [21,37,38,39]. Although the above results indicated a link between *MniDMRT11E* and gonadal maturation, the exact function of *MniDMRT11E* during gonadal maturation remains unclear. Therefore, we further investigated its function in *M. nipponense* by injecting *MniDMRT11E* dsRNA. *MniDMRT11E* mRNA expression was significantly inhibited seven days after the dsRNA injection in vivo compared to the control group, confirming that RNAi using dsRNA was an effective and valuable tool for studying specific gene functions by gene silencing. Meanwhile, *MniDMRT11E* RNAi caused a significant negative regulation of *VG* gene expression in the female ovary and hepatopancreas. However, *MniDMRT11E* RNAi resulted in significantly positively regulation of the *IAG* transcripts in the abdominal ganglia in males. In *M. nipponense*, the main sites of *VG* synthesis are the ovary and hepatopancreas. *VG* RNAi inhibited maturation of the ovary [38]. The relationship between *MniDMRT11E* and *MniVG* also explains the high expression of *MniDMRT11E* in the hepatopancreas(female) (Figure 4). In *M. rosenbergii*, silencing of *MrIAG* led to the arrest of testicular spermatogenesis and of spermatophore development in the terminal ampullae of the sperm duct, accompanied by hypertrophy and hyperplasia of the AGs. In addition, the sex reversal of male *M. rosenbergii* occurred through the silencing of a single *IAG*-encoding [4,36]. This also explains why the *MniDMRT11E* mRNA was visualized in glandular cells of AG (Figure 8). Considering the important involvement of *IAG* in crustacean males [4,5,6,7,8,36] and the vitally important involvement of *VG* in females [32,38,40], we hypothesized that *MniDMRT11E* may participate in this pathway, either directly or indirect upstream of *IAG*/*VG*. This result provides strong evidence for an important role of *MniDMRT11E* in promoting ovary maturity and inhibiting testis maturity. It is noteworthy that *MroDMRT11E* has a positive regulatory effect on *IAG* in patients with *M. rosenbergii*, but in *M. nipponense*, *MniDMRT11E* is the opposite. Crustaceans are less developed animals than vertebrates. Even in related species, the functions of the same gene among different species are different. The results of RNAi suggest that further research is needed to elucidate the function of *MniDMRT11E*.

In this study, we have identified a *DMRT* gene from *Macrobrachium nipponense*. These results suggested that the *MniDMRT11E* gene did not have dimorphic gene expression, but it could promote gonadal development, as well as embryogenetic expression patterns. The *MniDMRT11E* RNAi displayed negative regulation of the *VG* gene in the ovary and hepatopancreas and positive regulation of the *IAG* gene in abdominal ganglia. This study advances our understanding of the biological functions of the *MniDMRT11E* gene in *M. nipponense*.

## 4. Materials and Methods

### 4.1. Experimental Animals and Sampling

Our study does not involve endangered or protected species. This study was approved by the Institutional Animal Care and Use Ethics Committee of the Freshwater Fisheries Research Center, the Chinese Academy of Fishery Sciences (Wuxi, China, FFRC125, 26 August 2016). Healthy adult prawns, M. nipponense, were collected from Tai Lake in Wuxi, China (120°13′44″E, 31°28′22″N) in June 2017. The weight of each male prawn was 2.8±0.5g and the weight of each female was 1.8±0.5g. All prawns were transferred to an aquarium, cultured in an inflated freshwater pool in an indoor facility, and fed parudina twice per day.

### 4.2. Nucleotide Sequence and Bioinformatics Analysis

Total RNA was extracted from different adult prawn tissues using RNAiso Plus Reagent (TaKaRa, Japan) [21]. The design of degenerate primers (DMRTDF1, DMRTDR1, DMRTDR2) was based on the highly conserved amino-acid and nucleotide sequences of DM domains (*M. rosenbergii*, KC801044.1; *D. magna*, AB361069.1; *D. melanogaster*, NM_169202.1). Total RNA extracted from a testis was subjected to 5′and 3′ RACE cDNA syntheses using a 5′-full RACE Kit and 3′-full RACE Core Set Ver. 2.0 Kit (TaKaRa, Japan). Two-step PCR cloning with gene-specific primers (listed in Table 1) was carried out as described in [21]. The ORF Finder program (https://www.ncbi.nlm.nih.gov/orffinder/), and BLASTX and BLASTN programs (http://www.ncbi.nlm.nih.gov/BLAST/) were used to deduce amino acid sequences. The spatial structure was predicted by I-TASSER (https://zhanglab.ccmb.med.umich.edu/I-TASSER/). A phylogenetic tree was generated by Molecular Evolutionary Genetics Analysis (MEGA 5.1, http://www.megasoftware.net) by the neighbor-joining method with bootstrapping replications of 1000.

### 4.3. Tissue Expression Analysis by Quantitative Real-Time PCR

After one week of culture in the laboratory, the eyestalk, brain, heart, hepatopancreas, gill, muscle, ovary, and testis were dissected from mature prawns (*n* = 5). The development of embryos was divided into seven stages based on a study of Bai et al. [26] (from the unfertilized egg (UE) to the first day larvae after hatching (L1)). The samples were dissected separately, immediately frozen in liquid nitrogen, and stored at −80 °C until processed.

The procedures for RNA isolation and cDNA synthesis were as described previously [21]. The expression profiles of *MniDMRT11E* in different tissues were determined using qPCR assays (CWBIO, Beijing, China) [21,41]. The relative copy numbers of *MniDMRT11E* mRNA were calculated according to the 2^−ΔΔCT^ comparative CT method [42]. Beta actin was constantly smooth expressed in different developmental stages of prawns. Bestkeeper analysis and similar methods were performed for expression levels of beta actin [43]. Differences in expression levels were considered significant at *p* < 0.05.

### 4.4. Expression Profiles of DMRT11E in Ovarian Cycle

The determination of ovarian stage was based on the color of the oocytes, according to the criteria stated in [28]: Stage I (transparent), Stage II (yellow), Stage III (light green), Stage IV (dark green), and Stage V (gray). Ovarian samples were treated in the same way as other tissue. Then, we used qPCR to detect the expression level.

### 4.5. In Situ Hybridization (ISH)

ISH was performed on 4µ-thick formalin fixed paraffin-embedded ovary and testis sections using the Zytofast PLUS CISH implementation kit, after they were embedded in paraffin, as described in [21]. The slides were examined under a light microscope. The anti-sense and sense probes of the chromogenic in-situ hybridization study were designed by Primer5 software based on the cDNA sequence of *MniDMRT11E*. Both anti-sense and sense probes were hybridized with the slide. The anti-sense probe (5′-GTCUUCGCGUAGGACUUCGGCGAGUAUUCUGGAGUG-3′) was prepared for the experimental group, whereas the sense probe (5′-CACUCCAGAAUACUCGCCGAAGUCCUACGCGAAGAC-3′) was prepared for the control group.

### 4.6. RNA Interference of DMRT11E

Deliberate ds*DMRT11E* synthesis and preservation were performed according to Li et al. [21]. The template for *DMRT11E* dsRNA synthesis was prepared by the amplification of testis cDNA with the primers DMRT-iF and DMRT-iR (Table 1). Eighty healthy mature female (stage I) prawns and 80 healthy mature male prawns were respectively assigned to two groups. The experimental group (*n* = 40) was injected with *DMRT11E* dsRNA (4 μg/g of body weight). DEPC water was injected at an equal dose based on gram body weight in the control group (*n* = 40). All tissues (androgenic gland, hepatopancreas, and gonad) from each group were randomly collected on the seventh day after the injection and dissected, frozen immediately in liquid nitrogen, and stored at −80 °C until analysis. *VG* and *DMRT11E* mRNA expression in female tissue and *IAG* and *DMRT11E* mRNA expression in male tissue were investigated to detect the interference efficiency by qPCR on the seventh day after the injection (*n* = 4).

### 4.7. Data Analysis

All data are expressed as means ± standard. Statistical differences were estimated by one-way ANOVA followed by LSD and Duncan’s multiple range test in tissue distribution, embryo stages, and ovary cycle. A two-side t-test was used to compare expression levels in RNAi. All statistical analyses were performed using SPSS 20.0 (SPSS, Chicago, IL, USA).

## Figures and Tables

**Table 1:** Primers of sequence used.

Primer Name	Sequence (5′-3′)	Description
DMRTD-F1	TGCGCCMGRTGYMGRAAYCAYGG	For DM-domain RACE
DMRTD-R1	ARSGCSACYTGSGCSGCCATNAC	For DM-domain RACE
DMRTD-R2	TGSGCSGCCATNACCCKYTGCC	For DM-domain RACE
5′-RACE outer	ATTACCCGTTGCCTCT	For 5′-RACE
5′-RACE inner	ACCACCAGCAGGCAGTTG	For 5′-RACE
DMRT-5′R	GCTTCTTGTGCCCCTTCA	For 5′-RACE
3′-RACE outer	GGTGGTGTCCTGCCTGAA	For 3′-RACE
3′-RACE inner	CCTGAAGGGGCACAAGAA	For 3′-RACE
DMRT-3′F	CCTGAAGGGGCACAAGAA	For 3′-RACE
DMRT-qF	ACGACCTTAGTAGGATGGACAGT	For RT-PCR
DMRT-qR	GAGTGGAGGCAATAGAATGGGTA	For RT-PCR
β-actinF	TATGCACTTCCTCATGCCATC	For RT-PCR
β-actinR	AGGAGGCGGCAGTGGTCAT	For RT-PCR
DMRT-P	CACTCCAGAATACTCGCCGAAGTCCTACGCGAAGAC	Probe foe ISH
DMRT-iF	TAATACGACTCACTATAGGGCTTAGTGTCAGACGCGACCA	For DMRT dsRNA
DMRT-iR	TAATACGACTCACTATAGGGCTTCGGCGAGTATTCTGGAG	For DMRT dsRNA
IAG-qF	CGCCTCCGTCTGCCTGAGATAC	For RT-PCR
IAG-qR	CCTCCTCCTCCACCTTCAATGC	For RT-PCR
VG-qF	GAAGTTAGCGGAGATCTGAGGT	For RT-PCR
VG-qR	CCTCGTTGACCAATCTTGAGAG	For RT-PCR

## Abbreviations

*DMRT*	doublesex and mab-3 related transcription factor
*VG*	vitellogenin
*IAG*	insulin-like androgenic gland factor
*dsx*	doublesex
qPCR	quantitative real-time reverse transcription PCR
EM	eosinophilic matrix
E	eyestalk
BR	brain
H	heart
HE	hepatopancreas
G	gill
M	muscle;
O	ovary
T	testis
AG	abdominal ganglia
UE	unfertilized egg
CS	cleavage stage
BS	blastula stage
GS	gastrula stage
NS	nauplius stage
ZS	zoea stage
L1	the first day larvae after hatching
O I	undeveloped stage
O II	developing stage
O III	nearly-ripe stage
O IV	ripe stage
O V	spent stage
OG	oogonium
OC	oocyte
N	nucleus
CM	cytoplasmic membrane
Y	yolk granule
FC	follicle cell
FM	follicle membrane
CT	collecting tissue
SG	spermatogonium
SC	spermatocyte
SC1	primary spermatocyte
SC2	second spermatocyte
ST	spermatid
S	sperm
WE	wall epithelium
EM	eosinophilic matrix
VD	vas deferens
EB	the ejaculatory bulb
ORF	open reading frame
*M. nipponense*	*Macrobrachium nipponense*
*M. rosenbergii*	*Macrobrachium rosenbergii*
cDNA	complementary DNA
FFRC	freshwater Fisheries Research Centre
ISH	in situ hybridization
CISH	chromogenic in situ hybridization

## References

[1] Bureau of Fishery, Ministry of Agriculture, People’s Republic of China (2018). Fisheries Economic Statistics. China Fishery Yearbook.

[2] Burtis K.C., Baker B.S. (1989). *Drosophila* doublesex gene controls somatic sexual differentiation by producing alternatively spliced mRNAs encoding related sex-specific polypeptides. Cell.

[3] Martín I., Ruiz M.F., Sánchez L. (2011). The gene transformer-2 of Sciara (Diptera, Nematocera) and its effect on *Drosophila* sexual development. BMC Dev. Biol..

[4] Ventura T., Manor R., Aflalo E.D., Weil S., Raviv S., Glazer L., Sagi A. (2009). Temporal silencing of an androgenic gland-specific insulin-like gene affecting phenotypical gender differences and spermatogenesis. Endocrinology.

[5] Chung J.S., Manor R., Sagi A. (2011). Cloning of an insulin-like androgenic gland factor (IAG) from the blue crab, Callinectes sapidus: Implications for eyestalk regulation of IAG expression. Gen. Comp. Endocrinol..

[6] Ma K.Y., Lin J.Y., Guo S.Z., Chen Y., Li J.L., Qiu G.F. (2013). Molecular characterization and expression analysis of an insulin-like gene from the androgenic gland of the oriental river prawn, *Macrobrachium nipponense*. Gen. Comp. Endocrinol..

[7] Vega-Alpízar J.L., Alfaro-Montoya J., Hernández-Noguera L., Umaña-Castro R., Aflalo E.D., Sagic A. (2017). Implant recognition and gender expression following ampoule-androgenic gland implantation in *Litopenaeus vannamei* females (Penaeidae). Aquaculture.

[8] Guo Q., Li S., Lv X., Xiang J., Sagi A., Manor R., Li F. (2018). A Putative Insulin-like Androgenic Gland Hormone Receptor Gene Specifically Expressed in Male Chinese Shrimp. Endocrinology.

[9] Zhang E.F., Qiu G.F. (2010). A novel Dmrt gene is specifically expressed in the testis of Chinese mitten crab, *Eriocheir sinensis*. Dev. Genes Evol..

[10] Yu Y.Q., Ma W.M., Zeng Q.G., Qian Y.Q., Yang J.S., Yang W.J. (2014). Molecular Cloning and Sexually Dimorphic Expression of Two Dmrt, Genes in the Giant Freshwater Prawn, *Macrobrachium rosenbergii*. Agric. Res..

[11] Raymond C.S., Murphy M.W., O’Sullivan M.G., Bardwell V.J., Zarkower D. (2000). Dmrt1, a gene related to worm and fly sexual regulators, is required for mammalian testis differentiation. Genes Dev..

[12] Raymond C.S., Shamu C.E., Shen M.M., Seifert K.J., Hirsch B., Hodgkin J., Zarkower D. (1998). Evidence for evolutionary conservation of sex-determining genes. Nature.

[13] Artyom K. (2012). Dmrt, genes in the development and evolution of sexual dimorphism. Trends Genet..

[14] Erdman S.E., Burtis K.C. (1993). The *Drosophila* doublesex proteins share a novel zinc finger related DNA binding domain. The EMBO journal.

[15] Murphy M.W., Lee J.K., Rojo S., Gearhart M.D., Kurahashi K., Banerjee S., Loeuille G.A., Bashamboo A., McElreavey K., Zarkower D. (2015). An ancient protein-DNA interaction underlying metazoan sex determination. Nat. Struct. Mol. Biol..

[16] Zhang T., Zarkower D. (2017). DMRT proteins and coordination of mammalian spermatogenesis. Stem Cell Res..

[17] Hildreth P.E. (1965). doublesex, recessive gene that transforms both males and females of *drosophila* into intersexes. Genetics.

[18] Shen M.M., Hodgkin J. (1988). mab-3, a gene required for sex-specific yolk protein expression and a male-specific lineage in *C. elegans*. Cell.

[19] Picard M.A., Cosseau C., Mouahid G., Duval D., Grunau C., Toulza E., Allienne J.F., Boissier J. (2015). The roles of Dmrt (Double sex/Male-abnormal-3 Related Transcription factor) genes in sex determination and differentiation mechanisms: Ubiquity and diversity across the animal kingdom. Comptes Rendus Biol..

[20] Kato Y., Kobayashi K., Oda S., Colbourn J.K., Tatarazako N., Watanabe H., Iguchi T. (2008). Molecular cloning and sexually dimorphic expression of DM-domain genes in *Daphnia magna*. Genomics.

[21] Li F., Qiao H., Fu H., Sun S., Zhang W., Jin S., Jiang S., Gong Y., Xiong Y., Wu Y. (2018). Identification and characterization of opsin gene and its role in ovarian maturation in the oriental river prawn *Macrobrachium nipponense*. Comp. Biochem. Physiol. Part B Biochem. Mol. Biol..

[22] Kim B.M., Jeong C.B., Kim I.C., Yim J.H., Lee Y.S., Rhee J., Lee J. (2014). Identification of three doublesex genes in the monogonont rotifer *Brachionus koreanus* and their transcriptional responses to environmental stressor-triggered population growth retardation. Comp. Biochem. Physiol. Part B Biochem. Mol. Biol..

[23] Qiu G., Du N., Lai W. (1996). Studies on the male reproductive system of the freshwater praan, *Macrobrachium nipponense*: II. The morphology and ultrastructure of the sperm. Acta Zool. Sin..

[24] Qiao H., Xiong Y., Zhang W., Fu H., Jiang S., Sun S., Bai H., Jin S., Gong Y. (2015). Characterization, expression, and function analysis of gonad-inhibiting hormone in Oriental River prawn, *Macrobrachium nipponense* and its induced expression by temperature. Comp. Biochem. Physiol. Part A Mol. Integr. Physiol..

[25] Phoungpetchara I., Tinikul Y., Poljaroen J., Chotwiwatthanakun C., Vanichviriyakit R., Sroyraya M., Hanna P.J., Sobhon P. (2011). Cells producing insulin-like androgenic gland hormone of the giant freshwater prawn, *Macrobrachium rosenbergii*, proliferate following bilateral eyestalk-ablation. Tissue Cell.

[26] Bai H., Qiao H., Li F., Fu H., Jiang S., Zhang W., Yan Y., Xiong Y., Sun S., Jin S. (2016). Molecular and functional characterization of the vitellogenin receptor in oriental river prawn, *Macrobrachium nipponense*. Comp. Biochem. Physiol. Part A Mol. Integr. Physiol..

[27] Bellefroid E.J., Leclere L., Saulnier A., Keruzore M., Sirakov M., Vervoort M., De Clercq S. (2013). Expanding roles for the evolutionarily conserved Dmrt sex transcriptional regulators during embryogenesis. Cell. Mol. Llife Sci..

[28] Wexler J.R., Plachetzki D.C., Kopp A. (2014). Pan-metazoan phylogeny of the DMRT gene family: A framework for functional studies. Dev. Genes Evol..

[29] Zhu L., Wilken J., Phillips N.B., Narendra U., Chan G., Stratton S.M., Kent S.B., Weiss M.A. (2000). Sexual dimorphism in diverse metazoans is regulated by a novel class of intertwined zinc fingers. Genes Dev..

[30] Lourenço R., Lopes S.S., Saúde L. (2010). Left-Right Function of dmrt2 Genes Is Not Conserved between Zebrafish and Mouse. PLoS ONE.

[31] Seo K.W., Wang Y., Kokubo H., Kettlewell J.R., Zarkower D.A., Johnson R.L. (2006). Targeted disruption of the DM domain containing transcription factor Dmrt2 reveals an essential role in somite patterning. Dev. Biol..

[32] Wilder M.N., Okumura T., Tsutsui N. (2010). Reproductive mechanisms in Crustacea focusing on selected prawn species: Vitellogenin structure, processing and synthetic control. Aqua Biosci. Monogr..

[33] Yi W., Zarkower D. (1999). Similarity of DNA binding and transcriptional regulation by *Caenorhabditis elegans* MAB-3 and *Drosophila melanogaster* DSX suggests conservation of sex determining mechanisms. Development.

[34] Zhang X., Zha J., Wang Z. (2008). Influences of 4-nonylphenol on doublesex-and mab-3–related transcription factor 1 gene expression and vitellogenin mRNA induction of adult rare minnow (*Gobiocypris rarus*). Environ. Toxicol. Chem..

[35] Shukla J.N., Palli S.R. (2012). Doublesex target genes in the red flour beetle, *Tribolium castaneum*. Sci. Rep..

[36] Ventura T., Manor R., Aflalo E.D., Weil S., Rosen O., Sagi A. (2012). Timing sexual differentiation: Full functional sex reversal achieved through silencing of a single insulin-like gene in the prawn, Macrobrachium rosenbergii. Biol. Rreprod..

[37] Shechter A., Glazer L., Cheled S., Mor E., Weil S., Berman A., Bentov S., Aflalo E.D., Khalaila I., Sagi A. (2008). A gastrolith protein serving a dual role in the formation of an amorphous mineral containing extracellular matrix. Proc. Natl. Acad. Sci. USA.

[38] Bai H., Qiao H., Li F., Fu H., Sun S., Zhang W., Jin S., Gong Y., Jiang S., Xiong Y. (2015). Molecular characterization and developmental expression of vitellogenin in the oriental river prawn *Macrobrachium nipponense* and the effects of RNA interference and eyestalk ablation on ovarian maturation. Gene.

[39] French A.S., Meisner S., Liu H., Weckström M., Torkkeli P.H. (2015). Transcriptome analysisand RNA interference of cockroach phototransduction indicate three opsins and suggest a major role for TRPL channels. Front. Physiol..

[40] Subramoniam T. (2011). Mechanisms and control of vitellogenesis in crustaceans. Fisheries. Sci..

[41] Zhang Y., Jiang S., Xiong Y., Sun S., Qiao H., Jin S., Gong Y., Fu H. (2013). Molecular cloning and expression analysis of extra sex combs gene during development in *Macrobrachium nipponense*. Turk. J. Fish. Aquat. Sci..

[42] Livak K.J., Schmittgen T.D. (2001). Analysis of relative gene expression data using real-time quantitative PCR and the 2^−ΔΔCT^ method. Methods.

[43] Hu Y., Fu H., Qiao H., Sun S., Zhang W., Jin S., Jiang S., Gong Y., Xiong Y., Wu Y. (2018). Validation and evaluation of reference genes for quantitative real-time PCR in *Macrobrachium Nipponense*. Int. J. Mol. Sci..

